# Selection of Aptamers Specific for DEHP Based on ssDNA Library Immobilized SELEX and Development of Electrochemical Impedance Spectroscopy Aptasensor

**DOI:** 10.3390/molecules25030747

**Published:** 2020-02-09

**Authors:** Qi Lu, Xixia Liu, Jianjun Hou, Qiuxue Yuan, Yani Li, Sirui Chen

**Affiliations:** 1 Hubei Key Laboratory of Edible Wild Plants Conservation and Utilization, College of Life Sciences, Hubei Normal University, Huangshi 435002, China; lq18271691835@163.com (Q.L.); yqx1816752719@163.com (Q.Y.); yanilihbnu@163.com (Y.L.); 17371958850@163.com (S.C.); 2 Hubei Engineering Research Center of typical wild vegetable Breeding and Comprehensive Utilization Technology; Hubei Normal University, Huangshi 435002, China; 3 National Demonstration Center for Experimental Biology Education, Hubei Normal University, Huangshi 435002, China

**Keywords:** electrochemical aptasensor, ssDNA library immobilized SELEX, aptamer, di(2-ethylhexyl) phthalate, water sample

## Abstract

A selection of aptamers specific for di(2-ethylhexyl) phthalate (DEHP) and development of electrochemical impedance spectroscopy (EIS) aptasensor are described in this paper. The aptamers were selected from an immobilized ssDNA library using the systematic evolution of ligands by exponential enrichment (SELEX). The enrichment was monitored using real-time quantitative PCR (Q-PCR), and the aptamers were identified by high-throughput sequencing (HTS), gold nanoparticles (AuNPs) colorimetric assay, and localized surface plasmon resonance (LSPR). The EIS aptasensor was developed to detect DEHP in water samples. After eight rounds of enrichment, HTS, AuNPs colorimetric assay, and LSPR analysis indicated that four aptamers had higher binding activity, and aptamer 31 had the highest affinity (Kd = 2.26 ± 0.06 nM). The EIS aptasensor had a limit of detection (LOD) of 0.103 pg/mL with no cross-reactivity to DEHP analogs and a mean recovery of 76.07% to 141.32% for detection of DEHP in water samples. This aptamer is novel with the highest affinity and sensitivity.

## 1. Introduction

Di(2-ethylhexyl) phthalate (DEHP) is a kind of plasticizer that has toxic effects associated with its action as an androgen antagonist. It can lead to endocrine disorders and the reduction of human immunity [1,2]. However, DEHP is widely used as an additive to food packaging materials, which leads to its accumulation in food exceeding the national standards for its ability to migrate from food packaging to drinking water [3,4]. Therefore, it is necessary to continuously and effectively monitor the residue of DEHP.

At present, the methods for DEHP detection include analytical instrumentation, immunoassay, and biosensor. The commonly applied methods use analytical separation instruments. The limit of detection (LOD) of these methods is 0.05–10 mg/L for high-performance liquid chromatography (HPLC) [5,6,7], 0.01–0.1 ng/mL for high performance liquid chromatography–mass spectrometry (HPLC–MS) [8], 0.008–356 μg/mL for gas chromatography–mass spectrometry (GC–MS) [9], and 0.21 µg/L for mass spectrometry [10]. However, these methods are expensive, time-consuming, and have high LOD, which inhibit their application in the field for on-spot rapid detection. Alternatively, enzyme-linked immunosorbent assay (ELISA) has a LOD of 4.2 pg/mL [11], while the LOD is 3.9 pg/mL [12] for electrochemical aptasensor, 0.5 pg/mL for quantum dot aptasensor [13], and 8 pM for surface-enhanced raman spectroscopy (SERS) aptasensor [14]. The obvious advantages of these rapid-detection techniques are that they are fast, low-cost, and exhibit high selectivity and sensitivity in monitoring trace DEHP in samples. The quality of recognition molecules is critical for establishing these rapid-detection methods. However, the required antibodies used in ELISA always have issues, such as requiring more time for preparing monoclonal antibody and uneven quality for polyclonal antibody [15]. Therefore, aptamers may be the most suitable recognition molecules for the detection of DEHP.

An aptamer is a single-strand DNA (ssDNA) or RNA selected from DNA or RNA library using the systematic evolution of ligands by exponential enrichment (SELEX) [16]. A specific aptamer has many advantages, including short preparation time, a wide range of targets, and easy labeling and modification [17,18], which make it more appropriate than an antibody as a recognition molecule in biosensors [19]. There are several reports on the application of aptamers for the detection of small molecules, such as ractopamine [20], aflatoxin B1 [21], chloramphenicol [22], and ochratoxin A (OTA) [23]. Researchers have also successfully constructed aptamer-based biosensors to detect DEHP. Han [12] reported a group-specific aptamer to detect phthalic acid esters using an electrochemical biosensor, although the distinction of DEHP from phthalic acid esters was difficult. Later, a truncated aptamer specific for DEHP was reported by Lim [13], which also enabled establishing a quantum dot aptasensor. However, this detection technology was complex and was thus not used to detect real samples in their research. An aptasensor [14] based on SERS has also been developed with a LOD of 8 pM. However, this method included a series of procedures involving magnetic particles and silver nanoparticles with silica. Therefore, it is desirable to select aptamers that are specific for DEHP and design a direct DEHP detection method with improved sensitivity and affinity.

In this work, we isolated an aptamer specific for DEHP from immobilized ssDNA library and developed a direct electrochemical impedance spectroscopy (EIS) aptasensor. First, ssDNA with strong binding activity to DEHP was enriched by eight rounds of SELEX. Then, high-throughput sequencing (HTS), gold nanoparticles (AuNPs) colorimetric assay, and localized surface plasmon resonance (LSPR) were conducted to characterize the aptamer of highest affinity. Finally, an EIS aptasensor was established to directly detect DEHP residue in water samples.

## 2. Results and Discussion

### 2.1. Selection of Aptamers Specific for DEHP Based on ssDNA Immobilized SELEX

**Figure 1:**
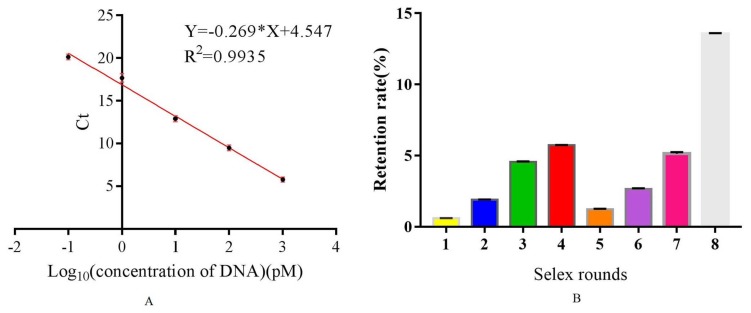
Q-PCR monitoring the process of selection. (**A**) The quantitative analytical curve of Q-PCR, (**B**) the retention rate for each round of selection.

A key step for aptamer selection involves monitoring the enrichment of the ssDNA library. An early report [24] has suggested that real-time PCR is the most simple and effective method to monitor the selection of aptamers specific for small molecules or other targets. Therefore, in this research, we used real-time PCR to monitor the enrichment of aptamers specific for DEHP. The analytical curve of quantitative PCR (Q-PCR) is shown in Figure 1A. The correlation coefficient *R*^2^ was 0.9935, indicating a good linear correlation between Ct (the corresponding cycle when the fluorescence reaches the fluorescence threshold) and log (concentration of template ssDNA). The retention rate (Figure 1B) was calculated according to the Ct value and analytical curve, and the calculation formulas of retention rate are mentioned in the supplementary information. The retention rate increased gradually from the first to the fourth round of selection but decreased from the fifth to the seventh round of selection due to the counter selection, which excluded most of the nonspecific binders. Further, from the fifth to the eighth round, the retention rate increased gradually because of specific gradual enrichment of the aptamer library. Particularly, the retention rate was higher in the eighth round of selection than in all the other rounds, indicating that the library was enriched specifically. Thus, the selection was stopped in the eighth round.

### 2.2. High-Throughput Sequencing and Sequence Analysis of the Enriched Library

The enriched library of the eighth round was sequenced by HTS technology, which showed a total of 20,000 different sequences. Compared with the commonly used method (cloning to a vector) [25], HTS is an effective method to obtain rich sequence information in a short time. Then, Three hundred sequences with a higher frequency of occurrence were selected, and the random area of 40 base sequences was analyzed by clustal X2 (Figure S1). Then, one hundred and twenty-nine sequences with the highest rate of homology were obtained by TreeView tool analysis (Figure S2), and their sequences were measured online on the mfold analysis platform to display one-ring region structure or more. Ninety-six sequences with several ring regions (≥2) were selected to identify binding activity. Each aptamer was truncated to 58 bases by removing 11 bases each from the 5′ and 3′ end constant regions from the original sequences (80 bases) for the next step.

### 2.3. Establishment of Gold Nanoparticles Colomertric Assay to Identify Active Aptamers

**Figure 2:**
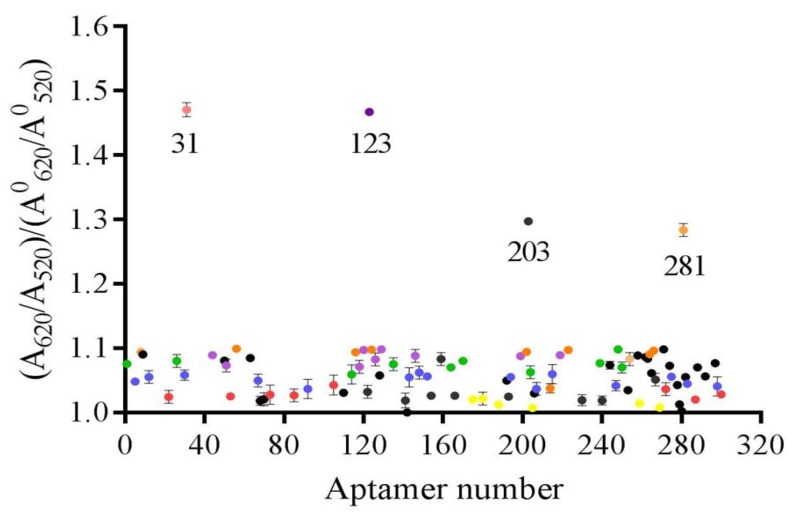
Preliminarily selection of active aptamers using the AuNPs colomertric assay.

The binding activity of candidate aptamers (96 aptamers) was initially determined using AuNPs, and the result is presented in Figure 2. The AuNPs were dispersed with the protection of the aptamer. When the added DEHP combined with the aptamer, the AuNPs lost the protection and aggregated due to the impact of the highly concentrated salt solution. The dispersed AuNPs appeared wine-red, and the aggregated AuNPs appeared blue. The characteristic peaks moved from 520 nm to 620nm. The ratio of A_620 nm_/A_520 nm_ between the experimental group and the blank group was close to 1 for most of the aptamers with low binding activity to DEHP. Interestingly, four aptamers had a ratio of >1.1, indicating that the A_620 nm_/A_520 nm_ value of the experimental group was greater than that of the blank group. This also showed the affinity between DEHP and aptamers, with a large number of combined complexes being produced. This is the first study to report a label-free, fast, and time-saving method for identification of the binding activity of aptamers specific for DEHP from an enriched library with a AuNPs colometric assay.

### 2.4. Characterization of Active Aptamers

**Figure 3:**
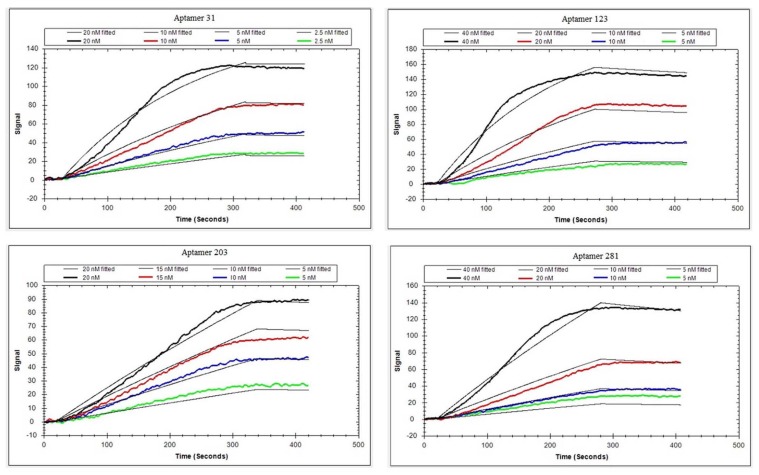
Binding kinetic curve of four aptamers interacting with di(2-ethylhexyl) phthalate (DEHP) by localized surface plasmon resonance (LSPR) assay.

**Figure 4:**
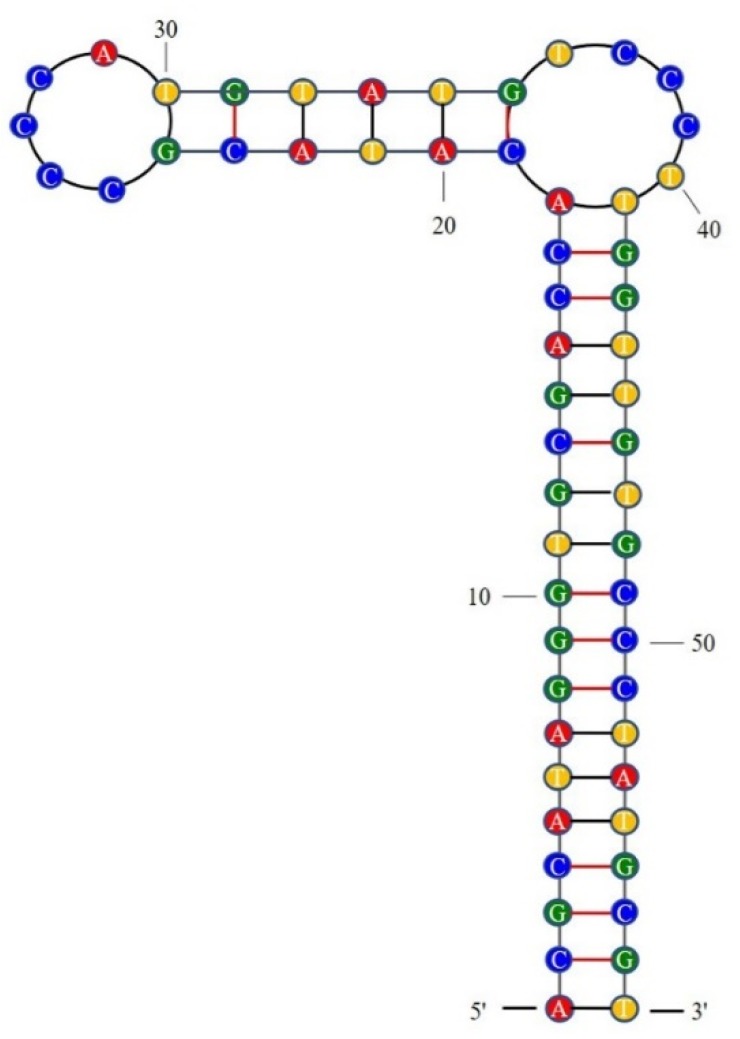
The secondary structure of aptamer 31.

Generally, electrochemistry [26], fluorescence [27], colorimetry [28], LSPR [29], and chemiluminescence [30] are applied to confirm the affinities of aptamers. LSPR is a label-free, money- and time-saving method for identification of aptamer affinity. The binding curves for four active aptamers in LSPR are presented in Figure 3. The signals increased when the aptamer concentration increased, indicating that the aptamer could bind with the DEHP on the surface of the chip. The detailed sequence information and affinity constants are noted in Table 1. Aptamer 31 had the highest affinity with Kd = 2.26 ± 0.06 nM, which was lower than that for all reported aptamers specific for small molecules. In a previous report, the affinity constants were beyond 10 nM for most aptamers [31,32]. Generally, LSPR is a powerful and sensitive tool for elucidation of the interaction between DNA and small molecules [33]. Therefore, aptamer 31, whose secondary structure is shown in Figure 4, was selected to develop DEHP detection methods.

### 2.5. Fabrication of Electrochemical Impedance Spectroscope Aptasensor

**Figure 5:**
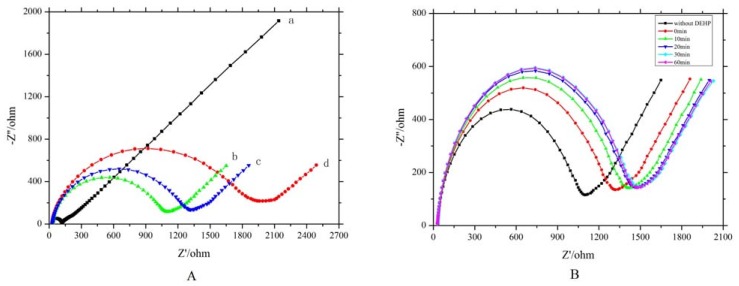
Electrochemical impedance spectroscopy (EIS) aptasensor. (**A**) Behavior of EIS aptasensor: (a) bare AuE, (b) aptamer 31 anchored on the gold electrode surface, (c) incubated with MCH, (d) incubated with DEHP. (**B**) Optimization of the incubation time between DEHP and aptamer.

EIS is highly sensitive to surface changes, which allows biorecognition events to be measured with a simple and label-free strategy for rapid bioanalysis. Here, we immobilized the biomolecule onto the electrode and then adopted the impedance method to follow the surface step-by-step electron transfer resistance (Rct) changes [34]. The behavior of the EIS aptasensor is shown in Figure 5A. The bare gold electrode displayed the smallest Rct value, indicating the fastest electrode reaction process of [Fe(CN)_6_]^3−^/^4-^ redox probes on conductive gold surface (curve a). The selected aptamer 31 was anchored on the gold electrode surface though Au–S bond, with sulfhydryl modified on the end of the 5′ end. According to the EIS spectrum, the Rct value obviously increased (curve b) due to the obstacles to electron transfer. Two main factors could be ascribed to the increase of the semicircle that indicated the increase of Rct. On the one hand, the negative-charged sugar-phosphate backbone of the aptamer on the electrode surface prevented [Fe(CN)_6_]^3−^/^4-^ from approaching the electrode surface due to strong electrostatic repulsion. On the other hand, the presence of the aptamer also partly blocked the electron/ion transport passageways between the electrode and the electrolyte. In addition, the immobilization of 6-mercapto-1-hexanol (MCH) on the electrode surface further blocked the electron/ion transport passageways, and the modified electrode with the aptamer and MCH thus gave a much higher Rct response (curve c). When the previously modified electrode was successively incubated with DEHP, a continuous augmentation in the semicircle was observed (curve d). This might be because the binding of DEHP to the aptamer resulted in a significant conformational change of the aptamer on the electrode surface. This change amplified the coverage of the aptamer on the electrode surface and therefore further impeded the electron transfer efficiency. The electron transfer resistance increased with increasing concentration of DEHP. This result is consistent with a previous report [35], indicating an increase in electron transfer resistance with conformational change of aptamer.

The optimal incubation time between DEHP and aptamer was explored to further improve the sensitivity, and the result is shown in Figure 5B. The EIS signal augmented during the incubation time from 0 to 30 min, but no distinct change was observed when the incubation time was beyond 30 min. Therefore, 30 min was deemed as the optimal incubation time between DEHP and aptamer. This result is in accordance with an earlier report that suggested 30 min incubation at room temperature [13].

### 2.6. Analytical Application of Sulfhydryl Modified Aptamer 31 in DEHP Detection Using EIS Aptasensor

**Figure 6:**
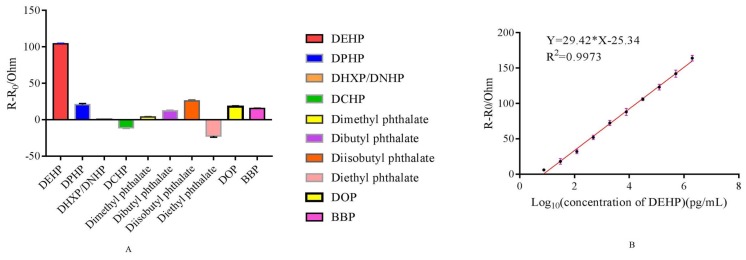
Specificity and sensitivity of EIS aptasensor. (**A**) Identification of the specificity of aptamer 31 against DEHP. The concentration of DEHP and analogs was 30.518 pg/mL. (**B**) Analytical curve of DEHP detection based on ultrasensitive EIS aptasensor.

The specificity of aptamer 31 was assessed, and the results are presented in Figure 6A. There was an obvious EIS signal when the detection target was DEHP, and the EIS signal decreased significantly when DEHP analogs were used as detection targets, clearly indicating that aptamer 31 had no cross-reactivity with DEHP analogs.

The analytical curve for the interaction is shown in Figure 6B. The EIS signal intensity augmented sharply with the increase in DEHP. Consequently, the increased EIS intensity Rct revealed a desirable linear correlation with the logarithmic value of the DEHP concentration, fitted as ΔR = 29.42 log(c) − 25.34 (*R*^2^ = 0.9973), where ΔR and c represent the increased EIS signal and the DEHP concentration, respectively. The LOD was 0.103 pg/mL, and the linear detection range was 7.629–2,000,000 pg/mL. The LOD was lower than that reported in previous studies for the detection of DEHP, which were listed in Table 2. Moreover, the LOD also proved to be below the national standard of 1.5 μg/mL, which was proposed in the GB9685-2008 for the use of additives for food containers and packaging materials. Therefore, the EIS aptasensor designed in this study exhibited high sensitivity and promising application for the detection of DEHP.

To explore the application of EIS aptasensor, we used aptamer 31 for water analysis, and the results are presented in Table 3. The average recoveries were observed from 76.07% to 141.32% with the relative standard deviation (RSD) ranging from 0.55% to 2.74%, indicating that the EIS aptasensor based on aptamer 31 is suitable for detection of DEHP residues in real samples.

## 3. Materials and Methods

### 3.1. Chemicals and Reagents

The DEHP standards and analogs were purchased from TMRM Ltd. (Beijing, China). Taq polymerase, dNTPs, and 2× TBE-urea buffer were purchased from Sangon Biotech Ltd. (Shanghai, China). Dynabeads™ MyOne™ Streptavidin T1 were purchased from Thermo Fisher Scientific Ltd. (Shanghai, China). HAuCl_4_·4H_2_O was purchased from Sinopharm Chemical Reagent Co., Ltd. (Shanghai, China). EvaGreen was purchased from Shanghai Open Biotechnology Ltd. (Shanghai, China). All other reagents were purchased from Sinopharm Chemical Reagents Ltd. (Shanghai, China). The aptamer library was synthesized by Sangon Biotech Ltd. (Shanghai, China). The primers in Table S1, according to our previous report [36], were synthesized by Nanjing Genscript Biotechnology Ltd. (Nanjing, China). The aptamers were synthesized from Suzhou Hongxun Biotechnology Ltd. (Suzhou, China). HTS was performed by Anhui Angputuomai Biotechnology Ltd. (Hefei, China). Secondary structures were calculated with mfold online bioinformatics platforms (http://unafold.rna.albany.edu/?q=mfold/DNA-Folding-Form). EIS aptasensor was performed on the CHI660E electrochemical workstation from Shanghai Chinstruments Ltd. (Shanghai, China). Milli-Q water was used for preparing all of the buffers and solutions.

### 3.2. Selection of Aptamers Specific for DEHP Based on ssDNA library immobilized SELEX

**Figure 7:**
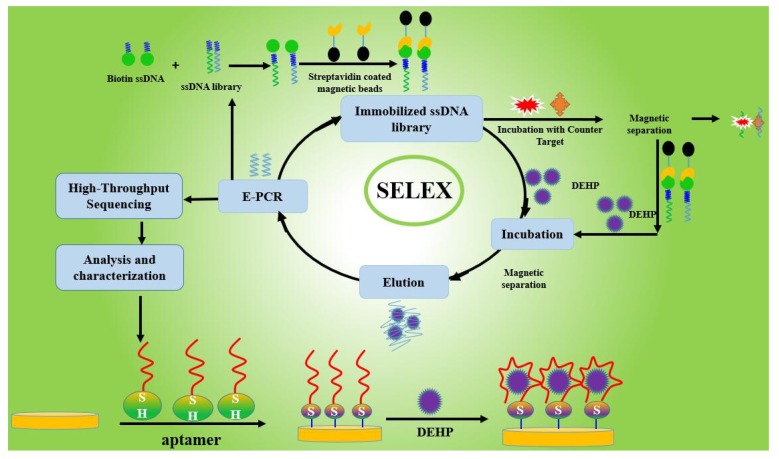
Graphical abstract.

The ssDNA library immobilized SELEX was established to select aptamers specific for DEHP, and the process of selection is shown in the graphical abstract (Figure 7). The 1.3 nM ssDNA library (5′-ATTGGCACTCCACGCATAGG(N)_40_CCTATGCGTGCTACCGTGAA-3′) was dissolved in 520 μL DPBS buffer (0.1 g CaCl_2_, 0.2 g KCl, 0.2 g KH_2_PO4, 0.1 g MgCl_2_.6H_2_O, 8 g NaCl, and 1.15 g Na_2_HPO_4_; 1 L, pH 7.5). A volume of 26 μL Biotin-P (100 μM in DPBS, pH 7.5) was added to the dissolved library at a molar ratio of 2:1 through the procedure of denaturation and renaturation at 95 °C for 10 min, 60 °C for 1 min, and 25 °C for 1 min. The obtained mixture was mixed with 700 μL streptavidin magnetic beads (7–10 × 10^9^ beads/mL) that were first washed 4 times with the DPBS buffer in the first round of selection. After incubating for 45 min at room temperature, the magnetic beads were washed 6 times and then incubated for 90 min with DEHP in 200 μL binding buffer to a final concentration of 100 μM. The supernatant was collected by magnetic separation. The magnetic beads were again washed with 200 μL binding buffer, and the supernatant was collected. The above supernatant was mixed as the eluent library with a volume of 400 μL in total.

### 3.3. Establishment of a Real-Time Quantitative PCR Method for the Monitoring Selection Process

The initial ssDNA library with a series of concentrations (16,000, 1600, 160, 16, and 1.6 pM) was used as a template in real-time Q-PCR. During this process, 2 μL of template was added into a 30 μL Q-PCR mix. The mix consisted of 1 μL Q-Forward at a concentration of 10 μM, 1 μL Q-Reverse at a concentration of 10 μM, 0.5 μL dNTP mix at a concentration of 10 mM, 3 μL 10×PCR buffer, 1 μL Taq DNA polymerase, and 1 μL EvaGreen, with sterile water added to make a final volume of 30 μL. The Q-PCR procedure (StepOnePlus purchased from ABI in the USA) was adopted with the condition of 95 °C for 2 min, 95 °C for 0.5 min, 60 °C for 0.5 min, and 72 °C for 0.5 min; there were 30 cycles in total. The analytical curve of Q-PCR was prepared using Ct value as an ordinate and logarithm DNA concentration of the template as an abscissa. The Q-PCR amplification was used to monitor the enrichment of libraries with the 2 μL eluent library as the template from each round of selection.

### 3.4. Preparation of Secondary Libraries

After each round of selection, all of the eluent library (398 μL template DNA) was mixed with 2 mL emulsion PCR mix that consisted of 10 μL FAM-Forward at a concentration of 100 μM, 10 μL polyA-Reverse at a concentration of 100 μM, 40 μL dNTP mix at a concentration of 10 mM, 200 μL 10× PCR buffer, 8 μL Taq DNA polymerase, and 1732 μL sterile water. Then, the 8 mL emulsifier (1 mL EM 90, 25 μL triton X-100, and 49 mL mineral oil) was added. After vortexing for 2 min, it was stood for 5 min. The PCR proceeded at 95 °C for 2 min, 95 °C for 1 min, 60 °C for 1 min, and 72 °C for 1 min for 25 cycles. The PCR product was concentrated with n-butyl alcohol. Then, it was mixed with 2× TBE-urea buffer and separated at denatured SDS-PAGE (400 V, 15 min). The separated fluorescent strip was cut off and boiled to isolate the secondary ssDNA library. It was dialyzed using a 1.5 mL tube cover and dialysis membrane in binding buffer overnight. The concentration of the secondary library was measured at 260 nm, and the secondary library was used to select specific aptamers in the next round of selection. The process of selection from the first to fourth round was the same, and the selections from the fifth to the eighth round were the counter selection with mixed analogs (diphenyl phthalate (DPHP), dihexyl phthalate (DHXP/DNHP), dicyclohexyl phthalate (DCHP), dimethyl phthalate, dibutyl phthalate, diisobutyl phthalate, diethyl phthalate, di-n-octylo phthalate (DOP), butyl benzyl phthalate (BBP)) and positive selection with DEHP. In the first round of selection, the amount of ssDNA library was 1.3 nM. However, in the other rounds, the amount of ssDNA library was 100 pM.

### 3.5. High-Throughput Sequencing and Sequence Analysis of the Enriched Library

The enriched library at the eighth round was sent to Anhui Angputuomai Biotechnology Co., Ltd. for HTS on an Illumina PE150 HTS platform. Three hundred sequences with higher frequency of occurrence were selected from the HTS results. Homologous comparison of these three hundred sequences was carried out using clustalX2 software. The comparison was then analyzed by TreeView tool version 1.6.6, and one hundred and twenty-nine sequences with a high homology rate were selected for further analysis. The secondary structure was predicted using mfold online bioinformatics platforms (http://unafold.rna.albany.edu/?q=mfold/DNA-Folding-Form). Ninety-six sequences with ≥2 ring regions and the high frequency of occurrence were selected. Each aptamer was cut with 11 bases each from the 5′ and 3′ constant regions, and the 58 bases remaining were then synthesized.

### 3.6. Determination of Binding Activity Between Aptamers and DEHP Using a Gold Nanoparticles Colomertric Assay

A AuNPs colorimetric assay was used to preliminarily determine the binding activity of candidate unmodified aptamers. AuNPs were prepared by reducing chloroauric acid with sodium citrate as in a previous report [37]. The AuNPs solution was centrifuged at 12,000 r/min and dissolved in ultrapure water. The concentration of AuNPs (8.76 nM) was determined using UV–vis spectroscopy according to a previously reported method [38]. Firstly, 50 µL/well of aptamers with a concentration of 0.4 μM were incubated with 1 μg/mL DEHP (50 µL/well) for 30 min. AuNPs (50 µL/well, 8.76 nM) were mixed and incubated for 30 min, and 1.0 M NaCl (10 µL/well) was then added at room temperature. The absorbance values at 620 nm (A_620 nm_) and 520 nm (A_520 nm_) were measured on an automatic microplate reader (I3X, Molecular Devices, USA). The binding activity of aptamers was determined by the ratio of A_620 nm_/A_520 nm_ between the experimental group (A) and the blank group (A_0_).

### 3.7. Affinity Analysis of Active Aptamers Using LSPR

**Figure 8:**
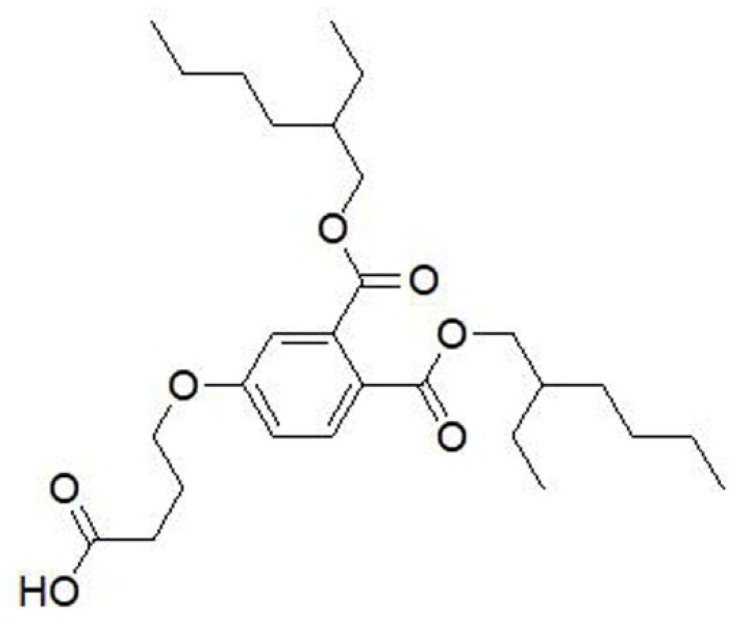
Structure of carboxy-modified DEHP.

The LSPR was used to determine affinity of aptamers on an OpenSPRTM (Nicoya Lifesciences, Waterloo, Canada). The specific steps were as follows. First, 100 μg DEHP modified with carboxyl group (Figure 8) was fixed to the NH_2_ chip through NH_2_ and COOH interaction and then blocked with 1 M ethanolamine (pH 8.5). After the baseline was adjusted with phosphate buffer solution (PBS, 29.2 g NaCl, 0.69 g NaH_2_PO_4_, 0.71 g Na_2_HPO_4_, 0.1 g MgCl_2_·6H_2_O; 500 mL, pH 7.0), aptamers (31, 123, 203, 281) diluted to different concentrations (2.5, 5, 10, and 20 or 5, 10, 20, and 40 nM) were allowed to bind with DEHP for 240s. The flowrate was 20 µL/min, and the chip was regenerated with 5–10 mM NaOH. The analysis software used in this experiment was TraceDrawer (Ridgeview Instruments ab, Sweden) using one-to-one analysis model [39].

### 3.8. Fabrication of EIS Aptasensor

The procedures of electrode pretreatment and modification were as follows. After polishing successively with 0.3 and 0.05 μm alumina, the gold electrodes (AuE, Φ = 3mm, CHI) were washed with four steps in 0.5 M NaOH, 0.5 M H_2_SO_4_, 0.1 M H_2_SO_4_ with 0.01 M KCl, and 0.05 M H_2_SO_4_ respectively, and then scanned by the cyclic voltammetry method. They were then thoroughly washed ultrasonically in ethanol and distilled water. Then, 2 μL of sulfhydryl modified aptamer (1 μM) was added into 2 μL activation solution (3 mg tris(2-carboxyethyl)phosphine (TCEP) in 100 μL PBS buffer) to open the disulfide bond formed by sulfhydryl groups between DNA for 1 h. After mixing with 10 mM PBS (pH = 7.0), the total volume reached 200 μL, and it was immediately suspended on the surface of the electrode to be incubated for 16 h. The surface of the electrode was then washed with PBS and subsequently immersed into a 0.5 mL MCH solution (2mM) for 5 h to avoid nonspecific binding of other molecules and then finally washed with PBS buffer. EIS was carried out using a CHI 660E electrochemistry workstation (Shanghai, China) in PBS containing 10 mM K_3_[Fe(CN)_6_]/K_4_[Fe(CN)_6_] (1:1) mixture and 0.5 M KCl as the supporting electrolyte with a conventional three-electrode system comprising a platinum wire as the auxiliary electrode, an Ag/AgCl as the reference electrode, and the modified AuE as the working electrode. The impedance spectra were recorded within the frequency range of 10^−2^–10^5^ Hz and AC amplitude of 5 mV. The surface behaviors of the electrode were studied, and the incubation time (0, 10, 20, 30, and 60 min) was optimized between the aptasensor and DEHP. The classical three-electrode system was used for EIS measurements. The Ag/AgCl electrode (saturated KCl), a platinum wire, and bare or modified gold electrode (3 mm in diameter) were used as reference electrode, counter electrode, and working electrode, respectively.

### 3.9. Specificity and Sensitivity Analysis of Aptamer 31

The pretreatment and modification of gold electrodes (AuE, Φ = 3mm, CHI) were carried out as mentioned above. The cross-reactivity (sulfhydryl modified aptamers incubated with DEHP analogs) was designed using DEHP and DEHP analogs (DPHP, DHXP/DNHP, DCHP, DOP, BBP, dimethyl phthalate, dibutyl phthalate, diisobutyl phthalate, diethyl phthalate) as detection targets with the concentration of 30.518 pg/mL at the optimal incubation time. The target was added to the supporting electrolyte of 10 mM K_3_[Fe(CN)_6_]/K_4_[Fe(CN)_6_] (1:1) mixture with 0.5 M KCl (pH 7.0) in PBS buffer. The impedance was detected, and the cross-reactivity was indicated by ΔR.

For the analytical curve, the pretreatment and modification of gold electrodes (AuE, Φ = 3mm, CHI) were carried out as mentioned above. The DEHP standard solution (1 μL) with different concentrations (7.629, 30.518, 122.07, 488, 1953, 7813, 31,250, 125,000, 500,000, and 2,000,000 pg/mL) was added to the supporting electrolyte of 10 mM K_3_[Fe(CN)_6_]/K_4_[Fe(CN)_6_] (1:1) mixture with 0.5 M KCl (pH 7.0) in PBS buffer. The impedance was detected, and ΔR was used as ordinate and log (DEHP concentration) as an abscissa.

### 3.10. Application of the EIS Aptasensor in Spiked Water Samples

The samples were collected from Qingshan Lake, tap water, and YB water. The DEHP standard was added to the water samples (negative samples) to obtain different final concentrations (30, 1950, and 500,000 pg/mL). Samples (1 μL) were added to the supporting electrolyte of 10 mM K_3_[Fe(CN)_6_]/K_4_[Fe(CN)_6_] (1:1) mixture with 0.5 M KCl (pH 7.0) in PBS buffer. The impedance was detected under the optimal conditions (aptamer concentration of 0.01μM, incubation time of 30 min), and the recovery was calculated.

## 4. Conclusions

Aptamers are functional molecules that can substitute antibodies in the development of rapid detection methods. In this research, the bottleneck problem was to select an aptamer specific for DEHP and to design a direct DEHP detection method for improving sensitivity and affinity. We proposed a rapid selection and identification strategy, which included Q-PCR monitoring, high- throughput sequencing, a AuNPs colomertric assay, and LSPR. After the eighth round of selection, the retention rate was about 14%. Four aptamers had high binding activity, and aptamer 31 had the highest affinity (Kd = 2.26 ± 0.06 nM). Then, the EIS aptasensor was used to detect water samples with a mean recovery of 76.07% to 141.32%. In this study, a novel aptamer specific for DEHP was selected, and a direct DEHP detection method based on an electrochemical aptasensor with ultrasensitivity was developed for low-cost, rapid, and sensitive detection. Most importantly, the rapid selection and identification strategy proposed in this study decreased the cost, complexity, and difficulty of the experiment. As a universal technical method, it can be used for the selection and identification of other aptamers specific for small molecules.

## 5. Patents

Liu, X.X.; Hou, J.J.; Lu, Q.; Yuan Q.X. An aptamer used to detect DEHP and its screening method and application. Patent 2019, application number: 201910547712.8

## Figures and Tables

**Table 1:** Sequence (5′-3′) and dissociation constant (Kd) of aptamer candidates.

Aptamer	Sequence (5′-3′)	Kd(nM)
31	ACGCATAGGGTGCGACCACATACGCCCCATGTATGTCCCTTGGTTGTGCCCTATGCGT	2.26 ± 0.06
123	ACGCATAGGGCAACCAGACCAGCCCCATCCCCATGTGACTTCTGTTTGGCCTATGCGT	5.33 ± 0.01
203	ACGCATAGGGCAAGACAAACTGCGCCATTCAGCATGCTGTTCGGGTTGGCCTATGCGT	2.68 ± 0.2
281	ACGCATAGGGTGTGCATCAGCAGTACCAACGACGTTGTGGTGTGCTCATCCTATGCGT	43 ± 0.7

**Table 2:** Comparison of available methods for analysis of DEHP.

Methods	Linear Range	Limit of Detection (LOD)	References
High-performance liquid chromatography	50–100,000 ng/mL	/	5–7
High-performance liquid chromatography–mass spectrometry	0.01–0.1 ng/mL	1 pg/mL	8
Raman spectroscopy	0.008–182nM	3.12 pg/mL	14
Mass spectrometry	5–1000 ng/mL	210 pg/mL	10
Enzyme-linked immunosorbent assay	10^−3–^10^3^ ng/mL	4.2 pg/mL	11
Multiresidue detection method based on aptamer	0.5–30 ng/mL	3.9 pg/mL	12
An ultrasensitive electrochemical method	7.629 pg/mL-2 µg/mL	0.103 pg/mL	This work

**Table 3:** Recovery results of DEHP added in different water samples with the aptasensor.

Samples	Spiked Concentration (μg·L^−1^)	Detected Concentration (μg·L^−1^)	RSD %	Recovery %
Tap water	0.03	0.031	2.74	101.77
	1.95	1.71	1.44	87.37
	500	462.59	1.57	92.52
Water from YB	0.03	0.043	1.69	141.32
	1.95	1.73	2.50	88.50
	500	488.44	2.54	97.69
Water from Qingshan Lake	0.03	0.026	2.15	85.70
	1.95	1.49	2.19	76.07
	500	481.22	0.55	96.24

## Supplementary Materials

Table S1. Primer sequences; Figure S1. The 300 sequences with a greater frequency of occurrence from high-throughput sequencing; Figure S2. Phylogenetic tree analysis of 300 sequences; Retention rate formulas.

## References

[1] Melnick R.L. (2001). Is peroxisome proliferation an obligatory precursor step in the carcinogenicity of Di (2-ethylhexyl) phthalate (DEHP)?. Environ. Health Perspect..

[2] Saillenfait A.M., Langonne I., Leheup B. (2001). Effects of mono-n-butyl phthalate on the development of rat embryos: In vivo and in vitro observations. Toxicol. Appl. Pharm..

[3] How C.M., Yen P.L., Wei C.C., Li S.W., Liao V.H.C. (2019). Early life exposure to di (2-ethylhexyl) phthalate causes age-related declines associated with insulin/IGF-1-like signaling pathway and SKN-1 in Caenorhabditis elegans. Environ. Pollut..

[4] Azevedo R., Oliveira N., Maia C., Verde I. (2019). Effects of di(2-etilhexil) phthalate on human umbilical artery. Chemosphere.

[5] Aignasse M.F., Prognon P., Stachowicz M., Gheyouche R., Pradeau D. (1995). A new simple and rapid HPLC method for determination of DEHP in PVC packaging and releasing studies. Int. J. Pharm..

[6] Deng L., Wu F., Deng N.S., Yang Y. (2005). Determination of trace DEHP in aqueous solution by solid phase microextraction coupled with high performance liquid chromatography. Fresen. Environ. Bull..

[7] Li B.P., Lin Q.B., Song H., Li L.L. (2008). Determination of DEHP and DNOP in PVC film by ASE-RP-HPLC. Chin. J. Appl. Chem..

[8] Pinguet J., Kerckhove N., Eljezi T., Lambert C., Moreau E., Bernard L., Boeuf B., Decaudin B., Genay S., Masse M. (2019). New SPE-LC-MS/MS method for the simultaneous determination in urine of 22 metabolites of DEHP and alternative plasticizers from PVC medical devices. Talanta.

[9] Cai Z.M., Zhang Q.L., Zhao W.H., Yang K.F., Wang L.P., Luo N., Tan L.T. (2003). GC-MS determination of phthalates (DBP and DEHP) dissolved from plastic bags. Phys. Test. Chem. Anal. B.

[10] Liang D.P., Fang Y.P., Liu W.J., Zhang H., Qiu Y.T., Dong Y.M., Ning Y. (2018). Direct determination of bis (2-ethylhexyl) phthalate in environmental water samples by electrospray extraction ionization mass spectrometry. Chin. J. Anal. Chem..

[11] Zhang M., Hong W., Wu X., Zhang Y., Li F., Zhao S.Q. (2015). A highly sensitive and direct competitive enzyme-linked immunosorbent assay for the detection of di-(2-ethylhexyl) phthalate (DEHP) in infant supplies. Anal. Methods UK.

[12] Han Y., Diao D.L., Lu Z.W., Li X.N., Guo Q., Huo Y.M., Xu Q., Li Y.S., Cao S.L., Wang J.C. (2017). Selection of group-specific PAE-binding DNA aptamers via rational designed target immobilization and applications for ultrasensitive and highly selective detection of PAEs. Anal. Chem..

[13] Lim H.J., Kim A.R., Yoon M.Y., You Y., Chua B., Son A. (2018). Development of quantum dot aptasensor and its portable analyzer for the detection of di-2-ethylhexyl phthalate. Biosens. Bioelectron..

[14] Tu D.D., Garza T.J., Coté L.G. (2019). A SERS aptasensor for sensitive and selective detection of bis(2-ethylhexyl) phthalate. Rsc Adv..

[15] Bird R.E., Walker B.W. (1991). Single chain antibody variable regions. Trends Biotechnol..

[16] Zhong W., Pu Y., Tan W.H., Liu J., Liao J., Liu B., Chen K., Yu B., Hu Y.L., Deng Y.Y. (2019). Identification and application of an aptamer targeting papillary thyroid carcinoma using tissue-SELEX. Anal. Chem..

[17] Shao N.S., Li S.H., Huang Y.P. (2006). New development of SELEX technology and aptamer research. Prog. Biochem. Biophys..

[18] Gong S., Wang Y.L., Wang Z., Sun Y.Y., Zhang W.B. (2018). Folding behaviors of purine riboswitch aptamers. Wuhan Univ. J. Nat. Sci..

[19] Shen R., Tang J.J., Zhang Z.Y., Guo L., Xie J.W. (2009). New magnetic beads-based enzyme linked aptamer colorimetric assay for trace amount protein detection. Chem. Res. Chin. Univ..

[20] Duan N., Gong W.H., Wu S.J., Wang Z.P. (2017). An ssDNA library immobilized SELEX technique for selection of an aptamer against ractopamine. Anal. Chim. Acta.

[21] Beheshti-Marnani A., Hatefi-Mehrjardi A., Es’haghi Z. (2019). A sensitive biosensing method for detecting of ultra-trace amounts of AFB1 based on “Aptamer/reduced graphene oxide” nano-bio interaction. Colloids Surf. B.

[22] Lu J.H., Tan S.Z., Zhu Y.Q., Li W., Chen T.X., Wang Y., Liu C. (2019). Fluorescent aptamer functionalized graphene oxide biosensor for rapid detection of chloramphenicol. Acta. Chim. Sin..

[23] Bianco M., Sonato A., De Girolamo A., Pascale M., Romanato F., Rinaldi R., Arima V. (2017). An aptamer-based SPR-polarization platform for high sensitive OTA detection. Sens. Actuat. B Chem..

[24] Luo Z.F., He L., Wang J.J., Fang X.N., Zhang L.Y. (2017). Developing a combined strategy for monitoring the progress of aptamer selection. Analyst.

[25] Lee A.Y., Ha N.R., Jung I.P., Kim S.H., Kim A.R., Yoon M.Y. (2017). Development of a ssDNA aptamer for detection of residual benzylpenicillin. Anal. Biochem..

[26] Challier L., Mavre F., Moreau J., Fave C., Schollhorn B., Marchal D., Peyrin E., Noel V., Limoges B. (2012). Simple and highly enantioselective electrochemical aptamer-based binding assay for trace detection of chiral compounds. Anal. Chem..

[27] Li J., Zhong X., Zhang H., Le X.C., Zhu J.J. (2012). Binding-induced fluorescence turn-on assay using aptamer-functionalized silver nanocluster DNA probes. Anal. Chem..

[28] Gopinath S.C.B., Lakshmipriya T., Awazu K. (2014). Colorimetric detection of controlled assembly and disassembly of aptamers on unmodified gold nanoparticles. Biosens. Bioelectron..

[29] Yoo S.M., Kim D.K., Lee S.Y. (2015). Aptamer-functionalized localized surface plasmon resonance sensor for the multiplexed detection of different bacterial species. Talanta.

[30] Li S.Y., Chen D.Y., Zhou Q.T., Wang W., Gao L.F., Jiang J., Cui H. (2014). A general chemiluminescence strategy for measuring aptamer-target binding and target concentration. Anal. Chem..

[31] Rangel A.E., Chen Z., Ayele T.M., Heemstra J.M. (2018). In vitro selection of an XNA aptamer capable of small-molecule recognition. Nucleic Acids Res..

[32] Wu Y.G., Zhan S.S., Wang L.M., Zhou P. (2014). Selection of a DNA aptamer for cadmium detection based on cationic polymer mediated aggregation of gold nanoparticles. Analyst.

[33] Zhang H.H., Yang S., Zhou Q., Yang L.K., Wang P.J., Fang Y. (2017). The suitable condition of using LSPR model in SERS: LSPR effect versus chemical effect on microparticles surface-modified with nanostructures. Plasmonics.

[34] Kharitonov A.B., Alfonta L., Katz E., Willner I. (2000). Probing of bioaffinity interactions at interfaces using impedance spectroscopy and chronopotentiometry. Electroanal. Chem..

[35] Zhou N.D., Luo J.B., Zhang J., You Y.D., Tian Y.P. (2015). A label-free electrochemical aptasensor for the detection of kanamycin in milk. Anal. Methods.

[36] Liu X.X., Lu Q., Chen S.R., Wang F., Hou J.J., Xu Z.L., Meng C., Hu T.Y., Hou Y.Y. (2018). Selection and identification of novel aptamers specific for clenbuterol based on ssDNA library Immobilized SELEX and gold nanoparticles colomertric assay. Molecules.

[37] Yao X., Ma X.D., Ding C., Jia L. (2016). Colorimetric determination of lysozyme based on the aggregation of gold nanoparticles controlled by a cationic polymer and an aptamer. Microchim. Acta.

[38] Haiss W., Thanh N.T.K., Aveyard J., Fernig D.G. (2007). Determination of size and concentration of nanoparticles from UV-Vis spectra. Anal. Chem..

[39] Lu Y., Xu X., Jiang T., Jin L., Zhao X.D., Cheng J.H., Jin X.J., Ma J., Piao H.N., Piao L.X. (2019). Sertraline ameliorates inflammation in CUMS mice and inhibits TNF-alpha-induced inflammation in microglia cells. Int. Immunopharmacol..

