# Supplementary material for: Selection of Aptamers Specific for DEHP Based on ssDNA Library Immobilized SELEX and Development of Electrochemical Impedance Spectroscopy Aptasensor
Source: Molecules. 2020 Feb 9;25(3):747. doi: 10.3390/molecules25030747 (PMC7038136; doi:10.3390/molecules25030747)
Supplement: Supplementary file 1 [file molecules-25-00747-s001.pdf]

Support information

Table S1. Primer sequences (Liu et al., 2018).

Name	Sequences (5′-3′)
Biotin-P	5′-CCTATGCGTGGAGTGCCAAT-3′-biotin
FAM-Forward	6-FAM-5′-ATTGGCACTCCACGCATAGG-3′
polyA-Reverse	5′-AAAAAAAAAAAAAAAAAAAAspacer18TTCACGGTAGCACGCATAGG-3′
Q-Forward	5′-ATTGGCACTCCACGCATAGG-3′
Q-Reverse	5′-TTCACGGTAGCACGCATAGG-3′

Figure S1. The 300 sequences with a greater frequency of occurrence from high-throughput sequencing sequences.

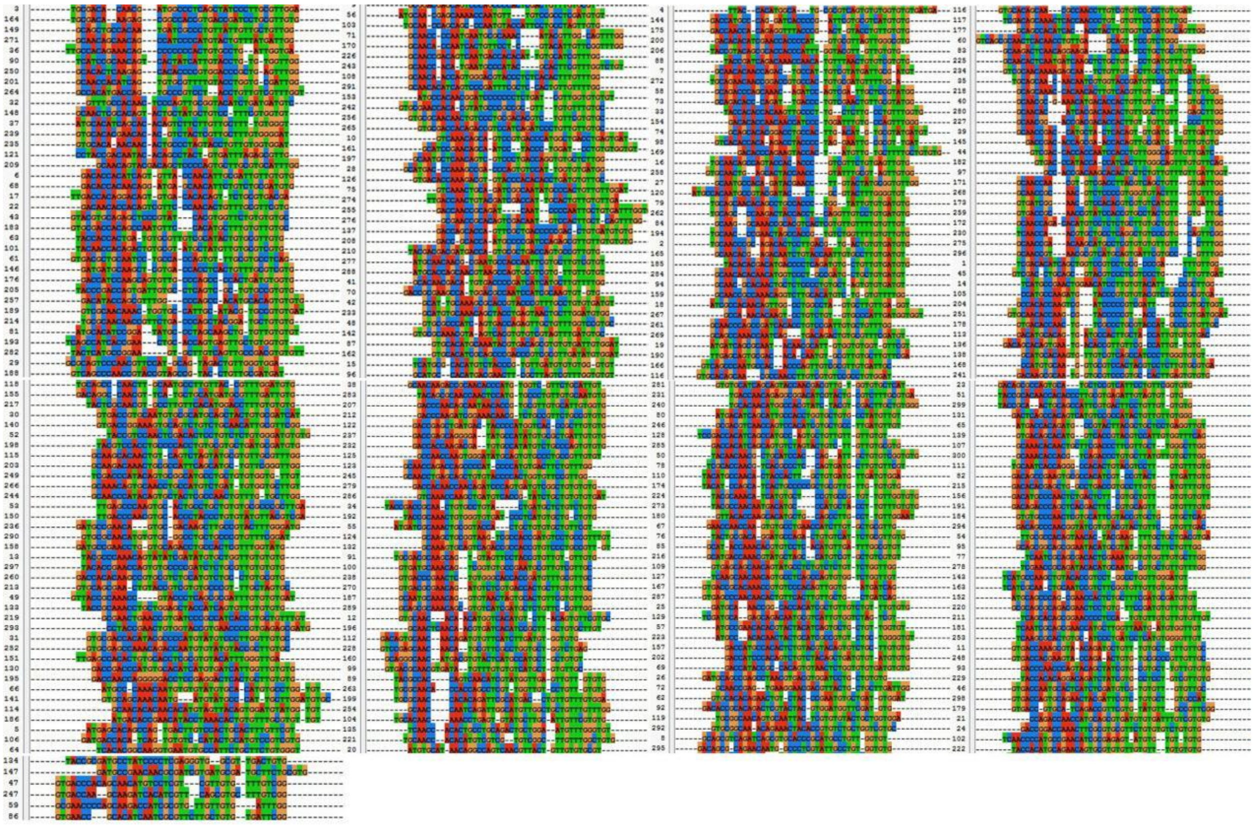

Figure S2. Phylogenetic tree analysis of 300 sequences.

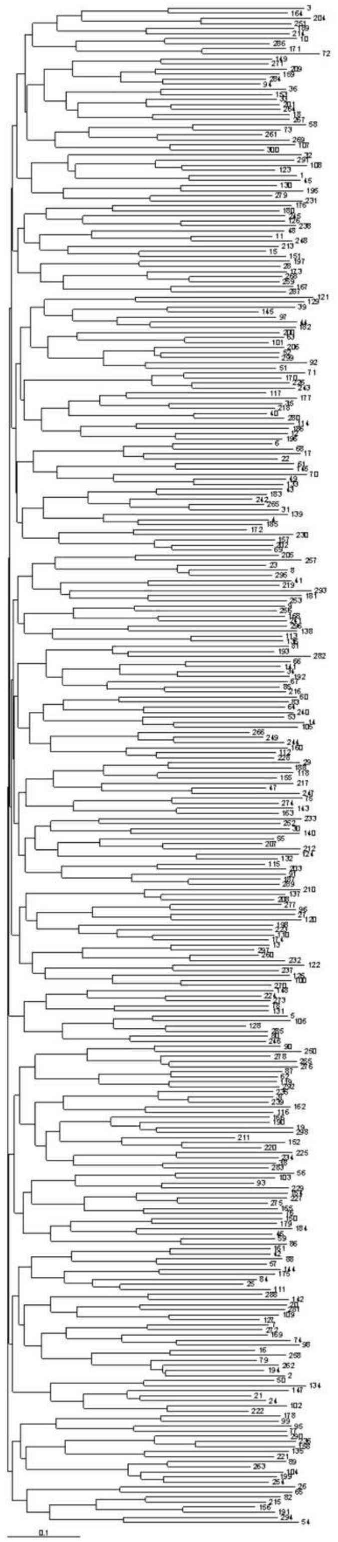

$$\text{Retention rate} = \frac{M_F}{M_I} \times 100\%$$

M_F: Molar of enriched ssDNA library of each round selection. We used Ct value (each round selection have corresponding Ct value.) and analytical curve to calculate the molar of enriched ssDNA library

M_I: Molar of initial ssDNA library of each round selection.
